# Chemical Compositions, Traditional Applications, and Biological Activities of *Gnaphalium affine* D. Don: A Comprehensive Review

**DOI:** 10.3390/molecules31071199

**Published:** 2026-04-04

**Authors:** Chen Ding, Yimiao Zhou, Lin Yang, Liquan Zhou, Zuowei Xiao

**Affiliations:** 1 Homologous Innovation Laboratory of Medicine and Food, Hunan University of Chinese Medicine, Changsha 410208, China; 20243738@stu.hnucm.edu.cn (C.D.); lauriechow@126.com (Y.Z.); zlq8067@126.com (L.Z.); 2 Hunan Engineering and Technology Research Center for Health Products and Life Science, Hunan University of Chinese Medicine, Changsha 410208, China; 3 School of Pharmacy, Hunan University of Chinese Medicine, Changsha 410208, China; 4 Xiangyin Campus, Xiangxing College, Hunan University of Chinese Medicine, Yueyang 414615, China; 5 Guizhou Institute of Crop Germplasm Resources, Guizhou Academy of Agricultural Sciences, Guiyang 550006, China; yanglin070809@163.com

**Keywords:** *Gnaphalium affine* D. Don, chemical compositions, traditional applications, biological activities

## Abstract

*Gnaphalium affine* D. Don (*G. affine*), a genus within the genus *Gnaphalium* of the Asteraceae family, is recognized as a significant medicinal resource. Phytochemical investigations identified various bioactive compounds in *G. affine*, including flavonoids, terpenoids, phenolic acids, alkaloids, and amino acids. These compounds exhibit a range of pharmacological activities such as antimicrobial, cough expectorant, antioxidant, and anti-inflammatory properties, as well as the regulation of lipid and glucose metabolism, reduction of uric acid levels, hepatoprotective effects, and anti-tumor activities. However, research concerning the chemical composition, biological activities, and potential applications of synthesized *G. affine* remains limited. In light of the growing interest in this species, the present paper aims to provide a comprehensive overview of the current research advancements related to the traditional applications, chemical constituents, and biological effects of *G. affine*. Additionally, this study will discuss future prospects for the development and application of *G. affine* to enhance its utilization in various fields.

## 1. Introduction

The concept of “medicine and food homology” represents a fundamental principle of traditional Chinese medicine (TCM), referring to natural substances that possess both nutritional and therapeutic functions due to their shared origin and intrinsic properties. This concept has been practiced for millennia in China and continues to play an important role in modern preventive medicine and functional food development [1]. The genus *Gnaphalium* comprises a group of medicinal and edible botanical resources of considerable interest. More than 200 species have been identified worldwide, of which 19 are distributed in China, mainly in regions south of the Yangtze River. Among them, *G. affine* is the most widely distributed and extensively utilized species, with the whole plant used for medicinal purposes [2].

*G. affine* has a long history of use in traditional medicine and is known by various vernacular names, including “Qingmingcai” [3]. Traditionally, it has been used to relieve cough and eliminate phlegm, as documented in classical medical texts such as *Mingyi Bielu (Records of Famous Physicians)* and *Bencao Gangmu (Compendium of Materia Medica)*. In addition, it is widely consumed as a traditional seasonal food in East Asia, reflecting its dual medicinal and nutritional value [4]. Phytochemical studies have demonstrated that *G. affine* contains diverse bioactive constituents, including flavonoids, phenolic acids, and terpenoids, which contribute to its multiple pharmacological activities, such as antibacterial, anti-inflammatory, antioxidant, hypoglycemic, and hypouricemic effects [5,6,7].

Despite its long-standing use and pharmacological potential, research on *G. affine* remains fragmented and largely descriptive, with the relationships between chemical constituents and biological activities poorly elucidated and the underlying molecular mechanisms insufficiently explored. To address these gaps, this review systematically analyzes 99 publications from 2000 to 2025, retrieved from Web of Science, PubMed, Scopus, and China National Knowledge Infrastructure (CNKI), focusing on traditional uses, phytochemistry, and biological activities. By integrating existing knowledge and highlighting research gaps, this work aims to provide a critical synthesis of *G. affine,* emphasizing mechanistic insights and supporting its further development as a functional botanical drug.

## 2. Botanical Characteristics

*G. affine* is an annual herb with slender white rhizomes, typically reaching 10–50 cm in height. The stem is solitary and erect, measuring 30–60 cm, and the leaves are ovate to lanceolate (2–5 × 0.5–1.5 cm) with a prominent midrib and acute apex. The flowers are yellow with brown central spots, 1–1.5 cm in diameter; the pedicels are short and robust, and the tepals are oblong to spatulate (0.8 cm), with a narrowed base and rounded apex. Perianth segments bear minute lobes and develop into cylindrical achenes (1–1.2 × 0.8–1 cm) lacking an apical beak, while the stamens are slender (0.6–0.8 cm). Inflorescences are dense and corymbose, 2–4 cm long and 2–3 cm in diameter. Flowering occurs from March to May, with fruit maturation from April to June [2]. *G. affine* commonly grows in low-altitude habitats, such as wastelands, hillsides, fields, and roadsides, and is widely distributed across China, as well as in North Korea, Japan, India, the Russian Far East, and Southeast Asia, including Indonesia, Malaysia, and Thailand (Flora of China).

## 3. Traditional Applications

*G. affine*, a member of the genus *Gnaphalium* (Asteraceae), holds a significant position in traditional Chinese medicine and folklore, with applications spanning medicinal, culinary, and cultural domains. In traditional medicine, the whole plant is used, and according to Chinese medicinal theory, it is considered flat and sweet in nature, belongs to the lung meridian, and possesses properties of moistening the lungs, resolving phlegm, and relieving cough and asthma. It is commonly administered as a decoction or tea for the treatment of respiratory conditions such as acute and chronic bronchitis and asthma, and can be applied externally to alleviate rheumatic arthralgia and bruises [8].

Beyond its medicinal value, *G. affine* is rich in flavonoids, terpenes, phenolic acids, alkaloids, and amino acids, conferring notable nutritional potential. Traditionally, young leaves are pounded into juice and mixed with glutinous rice flour to produce *Qingtuan* (emerald-green dumplings), a customary food consumed during the Qingming Festival for auspicious purposes. In regions such as Fujian and Taiwan, it is incorporated into local pastries or added to porridge and soups to impart a distinctive flavor [9].

In modern applications, *G. affine* continues to demonstrate considerable potential. Several medicinal products have been developed using its extracts, including Compound *Fo’er Cao Heji*, formulated for treating pediatric coughs and bronchitis [10]. Moreover, it has been innovatively utilized in the production of natural botanical fragrances and health-promoting teas [11]. The plant also serves as an eco-friendly dye for handmade paper in southern China, providing natural coloration for traditional crafts [12].

## 4. Chemical Compositions

### 4.1. Nutritional Components

As a medicinal and edible plant, *G. affine* is notable for its rich array of amino acids, vitamins, minerals, and bioactive compounds, contributing to both nutritional and pharmacological benefits. The plant contains 17 amino acids, including eight essential ones, with particularly high levels of glutamic acid and aspartic acid; total amino acid content reaches 18.25% in stems and 14.44% in flowers [13]. It is abundant in 25 elements, with potassium (K) content up to 38,508.15 mg/kg, alongside significant amounts of trace elements such as iron (Fe), manganese (Mn), and zinc (Zn). Vitamins A, C, E, and B2 are present in substantial quantities, with vitamin E notably reaching 2.31 mg/100 g. In terms of proximate composition, the moisture content ranges from 86% to 91%, total water-soluble sugars from 6.75% to 13.51%, crude cellulose from 20.30% to 31.90%, crude fat from 3.70% to 6.25%, and crude protein from 5.22% to 10.23%. Ash content varies between 0.84% and 1.30%, and vitamin C content ranges from 99 to 188 μg/g. Insoluble dietary fiber is the predominant fraction [14]. Collectively, these characteristics underscore the distinctive nutritional and therapeutic profile of *G. affine*.

### 4.2. Chemical Constituents

**Figure 1:**
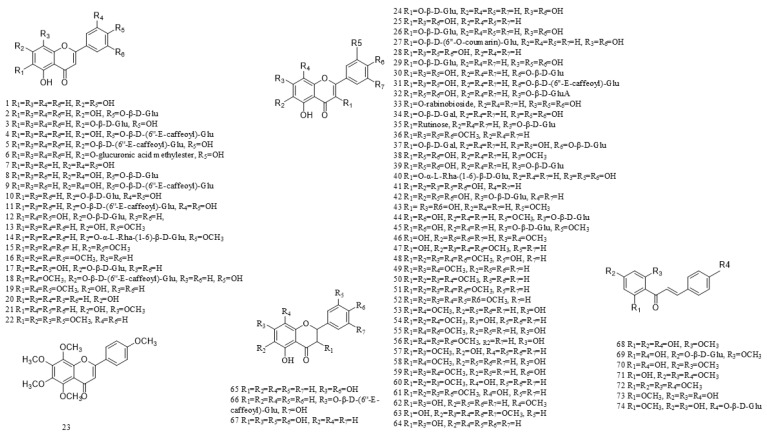
Chemical structure of flavonoids in *G. affine*.

**Figure 2:**
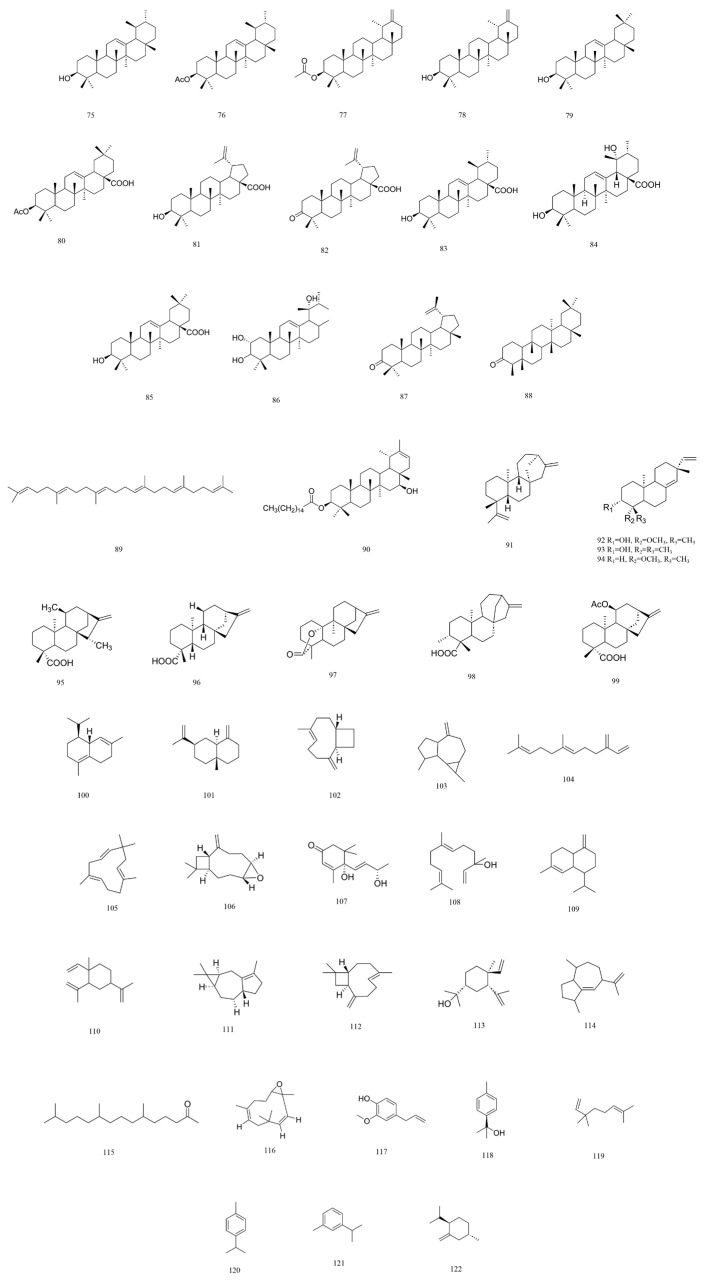
Chemical structure of terpenoids in *G. affine*.

**Figure 3:**
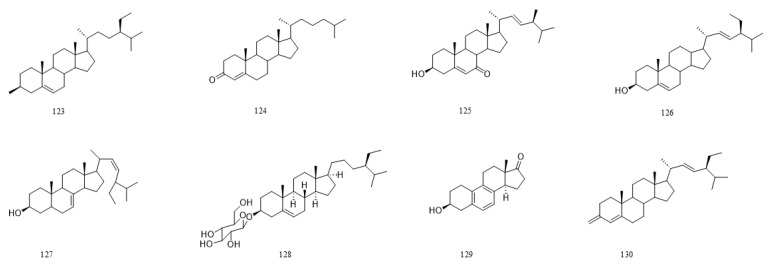
Chemical structure of phytosterols in *G. affine*.

**Figure 4:**
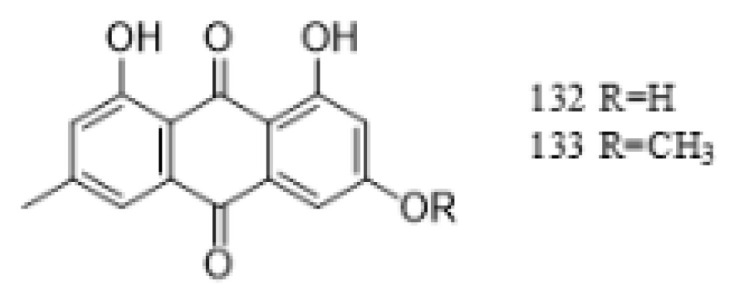
Chemical structure of quinone compounds in *G. affine*.

**Figure 5:**
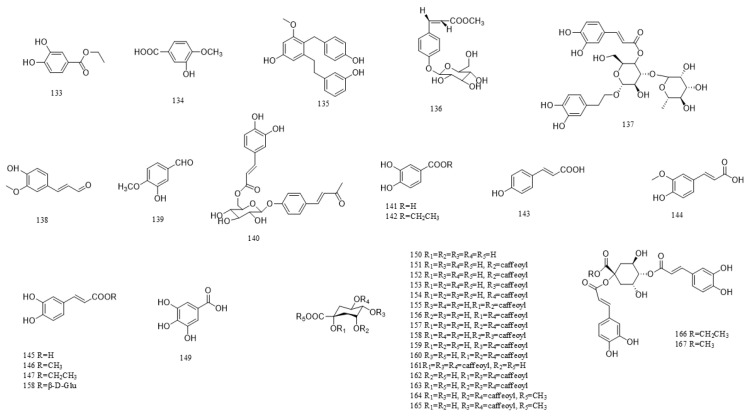
Chemical structure of phenolic acids and their derivatives in *G. affine*.

**Figure 6:**
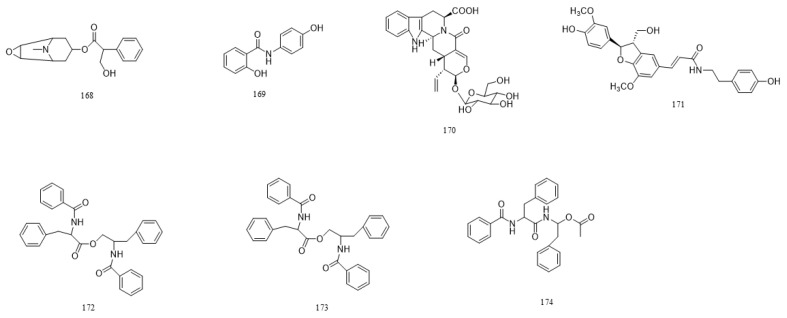
Chemical structure of alkaloids in *G. affine*.

**Figure 7:**
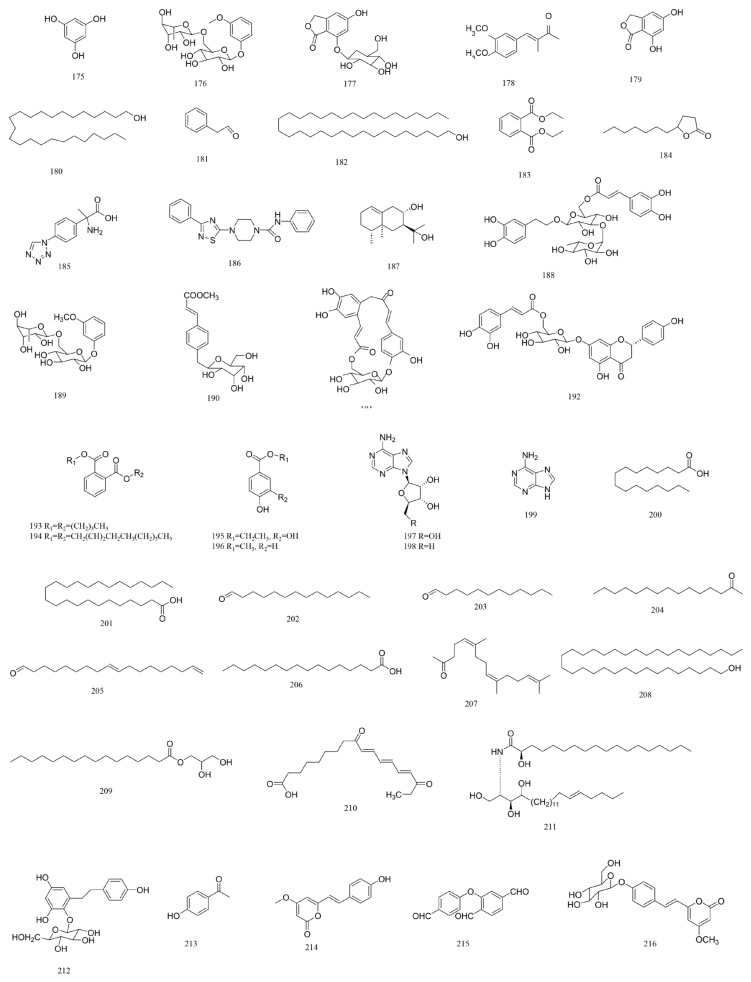
Chemical structures of other compounds in *G. affine*.

A total of 216 compounds have been identified from G. affine, encompassing flavonoids, terpenoids, phytosterols, anthraquinones, phenolic acids, alkaloids, and other bioactive constituents. These compounds exhibit diverse biological activities, including antioxidant, anti-inflammatory, and other pharmacological effects. The identified constituents are summarized in Table 1, with their structural representations shown in Figure 1, Figure 2, Figure 3, Figure 4, Figure 5, Figure 6 and Figure 7. Among them, flavonoids represent the predominant and most extensively studied class of active compounds.

#### 4.2.1. Flavonoids

A wide range of flavonoids has been isolated from *G. affine*, each exhibiting distinct pharmacological activities, such as antioxidant, anti-inflammatory, antibacterial, antitumor, and glucose-regulating effects. Based on chemical structure, they are classified into four major categories: flavones (1–23), flavonols (24–64), dihydroflavonoids (65–67), and chalcones (68–74), with flavonols being the most abundant. The detailed compounds are listed in Table 1, and their structures are illustrated in Figure 1.

#### 4.2.2. Terpenes

Terpenoids are a diverse class of natural compounds with multiple biological activities, including significant anti-inflammatory and antioxidant effects. Composed of isoprenoid (C_5_H_8_) units, they are biosynthesized through various pathways. *G. affine* contains a wide spectrum of terpenoids, among which triterpenoids are particularly prominent. Sesquiterpenes and monoterpenes are also commonly present, forming key components of the plant’s volatile oils. The specific compounds are detailed in Table 1, and their structures are shown in Figure 2.

#### 4.2.3. Phytosterols

Phytosterols, or plant sterols, are naturally occurring steroid compounds with diverse physiological functions, including cholesterol-lowering, antioxidant, anti-inflammatory, anticancer, and immunomodulatory effects. *G. affine* contains phytosterols such as β-sitosterol, stigmasterol, and taraxasteryl acetate. Detailed compounds are provided in Table 1, with structural representations illustrated in Figure 3.

#### 4.2.4. Anthraquinones

Anthraquinones are a class of quinones with significant pharmacological effects such as antibacterial, anti-inflammatory, and antitumor activities. The content of anthraquinones is relatively low in *G. affine*, and the anthraquinones identified so far are rhodopsin and rhodopsin methyl ether. The specific compounds are detailed in Table 1, with their structural representations illustrated in Figure 4.

#### 4.2.5. Phenolic Acids

Phenolic acids are organic compounds containing both phenolic hydroxyl and carboxylic acid groups, widely distributed in medicinal plants. They exhibit antioxidant, antimicrobial, anti-inflammatory, and anticancer activities. In *G. affine*, phenolic acids are predominantly caffeoylquinic acid derivatives. Detailed compounds are provided in Table 1, with their structures illustrated in Figure 5.

#### 4.2.6. Alkaloids

Alkaloids are nitrogen-containing organic compounds that occur widely in plants and display diverse pharmacological activities, including neuroinhibitory, muscle relaxant, antimicrobial, and antitumor effects. Seven alkaloid compounds have been isolated from *G. affine*. The specific compounds are listed in Table 1, with structural representations shown in Figure 6.

#### 4.2.7. Others

In addition to the above classes, other bioactive constituents have been identified in *G. affine*, including Gnaphaffine A, Gnaphaffine B, Tithoniamide B, and 4′-hydroxydehydrokawain [17]. The specific compounds are summarized in Table 1, with their structures illustrated in Figure 7.

## 5. Biological Activities

**Figure 8:**
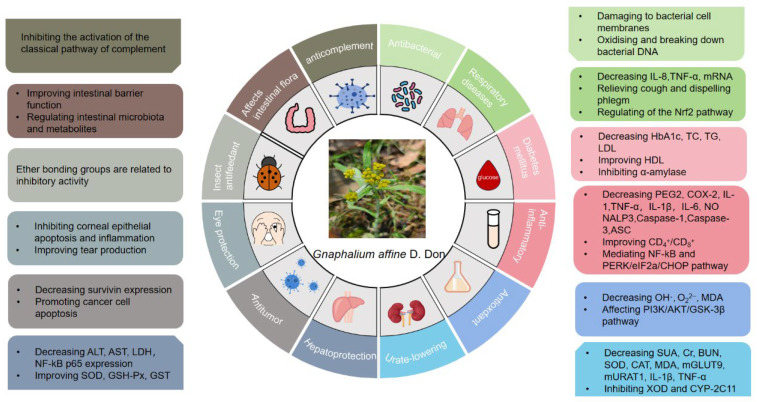
Pharmacological effects and mechanism of action of *G. affine*.

To date, numerous studies have elucidated the pharmacological properties of *G. affine* and its bioactive constituents. This plant exhibits diverse therapeutic effects, including antibacterial, antimicrobial, anti-inflammatory, antioxidant, anticomplement, hepatoprotective, and anticancer activities, and inhibition of xanthine oxidase. Flavonoids are recognized as the primary bioactive components, mediating antibacterial, anti-inflammatory, cough expectorant, antioxidant, and antitumor effects. A detailed summary of the pharmacological mechanisms is provided in Table 2 and Figure 8.

### 5.1. Antibacterial

Flavonoids are the primary contributors to the antibacterial activity of *G. affine*. Extracts containing 87.1% flavonoids exhibited inhibitory effects against four common pathogenic bacteria: *Staphylococcus aureus, Salmonella spp., Bacillus subtilis, and Escherichia coli* [37]. Among them, the flavonoid fraction demonstrated the strongest effect, with an inhibition zone diameter of 1.40 cm, while S. aureus was the most sensitive strain, exhibiting a minimum inhibitory concentration (MIC) of 1.25%, and relatively higher MIC values were observed for the other strains. Antibacterial efficacy correlated positively with flavonoid concentration (4.5–18 mg/mL), with higher concentrations producing stronger inhibition [38]. Moreover, flavonoids and their metal complexes exhibited greater activity against Gram-positive bacteria than against Gram-negative bacteria, likely due to differences in cell wall composition [39]. Mechanistically, flavonoids inhibit bacterial growth by disrupting cell membranes and inducing oxidative DNA degradation, with metal complexation enhancing protein and DNA binding, thereby improving antimicrobial efficacy compared with flavonoids alone [40].

Volatile oils extracted from *G. affine* also demonstrated broad-spectrum antimicrobial activity. At a concentration of 5 mg per disk, the oils inhibited *B. subtilis*, *S. aureus*, *B. cereus*, *B. lateralis*, *Saccharomyces cerevisiae*, *Aspergillus niger*, *Penicillium oryzae*, *A. oryzae*, and *A. flavus*, with inhibition zones ranging from 15.43 to 24.73 mm. Fungi, particularly yeasts, were more sensitive than bacteria. MIC values for bacterial strains ranged from 0.2 to 1.56 μg/mL, while minimum bactericidal concentrations (MBC) ranged from 0.39 to 3.13 μg/mL; MIC and MBC values for fungal strains were generally lower than those for bacteria [70]. These findings indicate that both flavonoids and volatile oils from *G. affine* provide multi-target antibacterial activity, with potency influenced by concentration, bacterial strain, and molecular interactions.

### 5.2. Respiratory Diseases

Traditionally, *G. affine* has been employed for managing respiratory ailments such as cough, phlegm, and sore throat. Its therapeutic effects involve inhibition of airway nerve excitability, modulation of immune responses, and facilitation of mucociliary clearance [71].

In preclinical studies, aqueous extracts significantly prolonged cough latency and reduced cough frequency in mice, while enhancing mucociliary clearance in guinea pig tracheal cilia [41]. Ethanolic extracts demonstrated efficacy in a rat model of COPD by reducing inflammatory cell infiltration in lung tissue, decreasing neutrophil and lymphocyte counts in bronchoalveolar lavage fluid, and downregulating inflammatory mediators, including IL-1β and interleukin-6 (IL-6), thereby mitigating pulmonary inflammation and tissue damage [42]. Western blot and RT-PCR analyses indicated modulation of oxidative stress pathways via upregulation of HO-1, Nrf2, and NQO1 expression [43].

Flavonoids isolated from *G. affine* also increased cough latency, reduced cough frequency, and promoted airway phenol red excretion in murine models. Additionally, they inhibited histamine-induced allergic asthma and prolonged the latency of asthma episodes, highlighting their potential in managing allergic respiratory conditions [44]. These data highlight the potential of *G. affine* and its flavonoids in modulating respiratory inflammation and improving airway function.

### 5.3. An-Inflammation

Inflammation is a fundamental protective response of the body to various stimuli, including pathogens such as bacteria and viruses, as well as physical injury [72]. *G. affine* and its flavonoid constituents exert multi-target anti-inflammatory effects, potentially beneficial in arthritis, pneumonia, and airway inflammation.

Studies have shown that alcoholic extracts of *G. affine*, administered at 0.80 g/kg, markedly reduced FCA-induced paw edema in rats, accompanied by decreased levels of inflammatory mediators, including PGE2, IL-1β, and TNF-α. The extract also appeared to restore the CD4^+^/CD8^+^ T-cell ratio by modulating T-cell subpopulations [45]. In GA models, the extract inhibited activation of the NALP3 inflammasome and reduced uric acid production through downregulation of NALP3/apoptosis-associated speck-like protein (ASC)/caspase-1 expression [46]. Further studies demonstrated that concentrations above 250 μg/mL decreased inflammatory cell infiltration in the swim bladder, while molecular docking suggested that compounds such as chlorogenic acid may target IL-1β and TNF-α, indicating multi-component mechanisms in antiviral pneumonia [47].

In osteoarthritis rat models, administration of the extract at 800 mg/kg improved pathological features of the knee joint, reduced levels of TNF-α, IL-6, and IL-1β, and modulated antioxidant enzyme activity, including malondialdehyde (MDA), SOD, and GSH-Px. Mechanistically, inhibition of the PERK/eIF2α/CHOP signaling pathway was observed [48]. In vitro, rat alveolar macrophages treated with 0–300 μg/mL of the extract retained 90–100% viability at concentrations up to 200 μg/mL, and lipopolysaccharide -induced inflammatory responses were attenuated via inhibition of NF-κB signaling, including p65 phosphorylation and nuclear factor of kappa light polypeptide gene enhancer in B-cells inhibitor, alpha degradation [73].

Moreover, the total flavonoids from *G. affine* also exhibited dose-dependent analgesic activity (50–100 mg/kg), mediated through suppression of macrophage-mediated inflammatory factor release [50]. Co-administration with antibiotics effectively alleviated airway inflammation in rats with COPD, likely via inhibition of apoptosis, reduction of matrix metalloproteinase activity, and modulation of the T helper cell 17/regulatory T-cell ratio [51].

### 5.4. Antioxidant

Oxidative stress causes damage to biomolecules, including proteins, lipids, and DNA, thereby compromising cellular integrity [74]. *G. affine* exhibits potent antioxidant activity, primarily attributed to its flavonoid and polyphenol constituents [75]. Quercetin, a major component, effectively scavenges free radicals and prevents H_2_O_2_-induced oxidative damage in Caco-2 cells [52]. Alcoholic extracts demonstrated concentration-dependent radical scavenging, achieving 42.59% and 28.76% clearance of hydroxyl radicals and superoxide anions at 2.0 mg, respectively [53].

Mechanistic studies indicate that *G. affine* extracts activate the PI3K/AKT/GSK-3β signaling pathway, upregulate SOD activity and Nrf2 expression, and modulate apoptosis-related proteins by downregulating B-cell lymphoma 2 (Bcl-2)-associated X protein and caspase-3 while upregulating Bcl-2, thereby mitigating H_2_O_2_-induced oxidative stress in HepG2 cells [54,55].

Total flavonoids from *G. affine* further enhance antioxidant capacity in vivo. In diabetic mice, they increased the activity of antioxidant enzymes, reduced lipid peroxidation (as indicated by MDA levels), and inhibited yolk lipoprotein peroxidation by 76.80% at 2.4 × 10^−2^ mg/mL, exceeding the efficacy of equivalent vitamin C concentrations [56,57]. IC_50_ values against DPPH, hydroxyl radicals, and sodium nitrite were 118.2, 16.3, and 17.8 μg/mL, respectively, demonstrating dose-dependent activity. Moreover, their antioxidant performance in soybean oil (IC_50_ = 33.4 μg/mL) was comparable to 0.02% TBHQ at 0.05% mass concentration [58]. Notably, the extracts facilitated AKT and GSK-3β phosphorylation without affecting AMP-activated protein kinase (AMPK), and this pathway activation was blocked by the PI3K inhibitor PX866. Additionally, the alcoholic extracts exhibited concentration-dependent antioxidant activity. [54].

At the cellular level, total flavonoids alleviated H_2_O_2_-induced death of HepG2 cells by reducing ROS and LDH release. Simultaneously, they increase SOD, CAT, and GSH levels, upregulate antioxidant genes (Nrf2, HO-1, and NQO1), and downregulate keap1 expression. Collectively, these effects establish an integrated antioxidant defense network that protects cells from oxidative damage [76,77].

### 5.5. Diabetes Mellitus

Diabetes mellitus is a prevalent metabolic endocrine disorder characterized primarily by an absolute or relative deficiency in insulin secretion. This insufficiency disrupts glucose metabolism, resulting in pronounced symptoms of hyperglycemia [78]. *G. affine* exhibits significant hypoglycemic and hypolipidemic effects, primarily due to flavonoids and polyphenols. These compounds act via inhibition of pancreatic α-amylase, enhancement of antioxidant capacity, and regulation of glucose and lipid metabolism.

Specifically, α-amylase inhibition reduces the breakdown and absorption of dietary starch glycosides [79]. The extract and active constituents, including 3,5-O-caffeoylquinic acid, 3,4-O-caffeoylquinic acid, and 2′,4,4′-trihydroxy-6′-methoxy-chalcone-4′-O-*β*-D-glucose, exhibited inhibition rates of 32.37%, 84.53%, 63.07%, and 71.22% at 1.67 mg/mL, with IC_50_ values of 2.71, 0.90, 1.28, and 1.16 mg/mL, respectively, and 2′,4,4′-trihydroxy-6′-methoxy-chalcone-4′-O-*β*-D-glucose acted via reversible non-competitive inhibition [60]. Moreover, flavonoids improved lipid metabolism by inhibiting cholesterol and triglyceride synthesis while promoting their breakdown. In diabetic mice, flavonoids improved glucose tolerance, reduced glycated serum protein, lowered total cholesterol, triglyceride, and LDL, and increased HDL and liver glycogen, highlighting their potential in mitigating cardiovascular risks [61].

### 5.6. Lowering UA

XOD plays a pivotal role as a key enzyme in the purine metabolic pathway, catalyzing the oxidation of purines to generate uric acid while simultaneously producing superoxide anion radicals as by-products [80,81]. Effective inhibition of XOD activity has been shown to reduce excessive uric acid production at its source and mitigate tissue damage induced by free radicals, a mechanism well-documented in previous studies [82]. Notably, *G. affine* effectively reduces uric acid levels through dual mechanisms: XOD inhibition and superoxide anion scavenging [83].

Alcoholic and solvent extracts suppressed MDA formation, restored antioxidant capacity, and modulated uric acid metabolism via XOD inhibition, with ethyl acetate fractions being most potent [62]. In particular, key compounds, including ethyl 1,4-di-O-caffeoylquinic acid (IC_50_ = 11.94 μM), (-)-methyl 1,4-di-O-caffeoylquinic acid (IC_50_ = 15.05 μM), luteolin, and luteolin-4′-O-glucoside, demonstrated potent XOD inhibition, enhanced uric acid excretion, and ameliorated renal impairment in murine models [63,84]. Moreover, co-administration of *G. affine* extract with Benzbromarone (BBR) was found to enhance anti-HUA activity while mitigating BBR-induced hepatotoxicity, suggesting a promising approach for HUA and gout therapy [64]. Overall, *G. affine* exerts multi-targeted effects against HUA and gout, and further characterization of its active components may inform novel therapeutic strategies.

### 5.7. Hepatoprotection

Recent studies have demonstrated that the alcoholic extract of *G. affine* confers significant protection against chemically induced liver injuries in rodent models. In CCl_4_-induced hepatotoxicity, alcoholic extracts restored liver architecture, attenuated hepatocellular damage, and reduced necrosis [85]. Similarly, total flavonoids (75–300 mg/kg/day for 30 days) decreased MDA levels and increased GSH and SOD activity in alcohol-induced liver injury [65]. In HIRI models, extracts at 0.4–1.6 g/kg/day for 28 days reduced hepatocellular injury, lowered serum ALT, AST, and LDH, and elevated SOD, GSH-Px, and GST activities. NF-κB, p65 expression was downregulated, indicating antioxidant and anti-inflammatory mechanisms [86].

Moreover, in APAP-induced acute liver injury, hepatocyte ferroptosis plays a pivotal role by increasing intracellular free iron and ROS, thereby exacerbating oxidative stress and cell death [87,88,89]. Treatment with total flavonoids of *G. affine* (100–400 mg/kg/day for 14 days) upregulated the expression of Nrf2, HO-1, SLC7A11, and GPX4, while downregulating Keap1. High-dose flavonoid treatment notably outperformed the positive control bifendate, indicating that *G. affine* inhibits ferroptosis and ameliorates APAP-induced acute liver injury through modulation of ferroptosis-related signaling pathways [66].

### 5.8. Antitumor

Previous studies have confirmed that *G. affine* exhibits multi-targeted antitumor effects, including modulation of oxidative stress, free radical scavenging, and apoptosis induction in tumor cells.

In liver cancer mouse models, aqueous extracts reduced tumor incidence in a dose-dependent manner (high: 40%, medium: 62.5%, low: 80%, and control: 100%) [67]. Mechanistically, antitumor activity involved induction of apoptosis, regulation of oxidative stress, and free radical scavenging in tumor cells. Similarly, in bladder and lung cancer models, aqueous extracts promoted apoptosis, decreased survivin expression, and exerted concentration-dependent effects. These findings indicate broad-spectrum anticancer potential mediated via oxidative stress modulation, apoptosis induction, and free radical scavenging [68].

### 5.9. Eye Protection

G. affine is rich in polyphenols with cytoprotective and anti-inflammatory properties. Among dicaffeoylquinic acids, 1,5-dicaffeoylquinic acid showed the strongest protective effect against dehydration-induced injury in human CECs. Topical application alleviated ocular inflammation and outperformed crude extracts and commercial dry eye treatments, such as Restasis^®^ and Hyalein mini 0.1%^®^ [69]. Mechanistically, protection involves inhibition of corneal epithelial apoptosis, stimulation of tear secretion, and suppression of inflammatory responses, suggesting potential therapeutic applications for ocular surface disorders, including dry eye syndrome.

### 5.10. Other Bioactivities

Beyond the above effects, *G. affine* extracts display insect-repellent properties [22]. Additionally, these extracts have been shown to enhance intestinal barrier function, modulate gut microbial communities and metabolites, and effectively mitigate high-fat diet-induced obesity [90]. Furthermore, luteolin demonstrates significant inhibitory activity on complement systems in vivo, with luteolin 4′-O-*β*-D-(6′-E-caffeoyl)-glucopyranoside exhibiting the highest potency, characterized by IC_50_ values as low as 0.045 ± 0.005 mg/mL [91].

### 5.11. Comparative Analysis with Other Medicinal and Edible Plants

The medicinal and edible plant *G. affine* shares several bioactivities with other well-known functional foods, yet exhibits distinct phytochemical and pharmacological characteristics that warrant comparative consideration. For instance, *Taraxacum officinale* (*Taraxacum officinale*) is rich in phenolic acids and sesquiterpene lactones, demonstrating notable hepatoprotective, diuretic, and anti-inflammatory effects [92,93]. *Houttuynia cordata* (*Houttuynia cordata)* contains flavonoids and volatile oils, and is widely used for its antimicrobial and anti-inflammatory properties, particularly in respiratory infections [94,95]. *Lonicera japonica* (honeysuckle) is valued for its broad-spectrum antibacterial and antiviral activities, attributed to chlorogenic acids and luteolin derivatives [96].

Compared with these plants, *G. affine* exhibits several unique features. First, its flavonoid profile is exceptionally diverse, with over 60 flavonoids identified, including characteristic compounds such as gnaphaliin A and B and 5,7-dihydroxy-3,6,8-trimethoxyflavone, which are less commonly reported in other medicinal herbs [91]. Second, *G. affine* demonstrates a remarkable combination of uric acid-lowering and anti-gout activities through dual inhibition of xanthine oxidase and modulation of renal urate transporters (mGLUT9 and mURAT1) [63], a mechanism less prominently documented in dandelion or honeysuckle. Third, its polysaccharide fraction contributes significantly to antioxidant and immunomodulatory activities, complementing the flavonoid-mediated effects [53,97]. Fourth, the plant’s safety profile, with an estimated LD_50_ exceeding 20.36 g/kg, compares favorably with many medicinal herbs and supports its long-standing use as a food ingredient [98].

From a therapeutic perspective, *G. affine* aligns with the growing interest in multi-target botanical drugs, particularly for chronic inflammatory conditions and metabolic disorders. While plants such as H. cordata are preferentially used for acute respiratory infections, *G. affine* offers broader applicability, spanning respiratory diseases, hyperuricemia, metabolic syndrome, and liver injury. Notably, its traditional use as a seasonal food (Qingtuan) positions it uniquely for functional food development, a dimension less emphasized for the comparator species.

In summary, while *G. affine* shares common bioactive classes with other medicinal and edible plants, its distinctive flavonoid composition, combined uricosuric and XOD-inhibitory activities, favorable safety profile, and established culinary use collectively distinguish it as a versatile resource for both pharmaceutical and nutraceutical applications. Comparative phytochemical and pharmacological studies across these species are warranted to further elucidate their respective strengths and therapeutic niches.

## 6. Safety

Recent toxicological investigations have demonstrated that *G. affine* exhibits minimal toxicity. In preclinical studies, oral administration of the extract at 20 mL/kg/day for 14 days caused no mortality or organ pathology, with an estimated LD_50_ exceeding 20.36 g/kg, classifying it as practically non-toxic; in a 28-day subacute study, doses of 25–100 mL/kg produced dose-dependent increases in spleen and thymus mass in female mice, yet all hematological, biochemical, and histopathological parameters remained within normal ranges, suggesting immunomodulatory rather than toxic effects [98]. Collectively, these findings indicate that *G. affine* is practically non-toxic at the tested doses. However, current toxicological studies remain limited, and long-term safety as well as human data are largely lacking.

## 7. Conclusions and Future Prospects

G. affine, a medicinal and edible plant of the Asteraceae family, exhibits a broad spectrum of pharmacological activities, including antibacterial, anti-inflammatory, antioxidant, hepatoprotective, hypoglycemic, antitumor, as well as traditional effects such as antitussive, expectorant, uric acid-lowering, and antihypertensive activities. Its bioactivities are primarily attributed to flavonoids, polyphenols, and dicaffeoylquinic acids, and its unique chemical profile, particularly the presence of compounds such as Gnaphalin and 4,4′,6′-trihydroxy-2′-methoxychalcone, distinguishes it from other Gnaphalium species. The dual role of *G. affine* as both a traditional herbal medicine and a dietary resource highlights its potential for functional food and nutraceutical development, as well as its relevance in disease prevention and health promotion. However, most current evidence is derived from in vitro and animal studies, and high-quality clinical validation remains limited.

Future research should focus on: (I) comprehensive toxicological profiling, including long-term safety, reproductive toxicity, and contaminant risk assessment; (II) mechanistic studies using integrated multi-omics approaches and high-throughput screening to identify novel bioactive compounds and clarify structure–activity relationships; (III) pharmacokinetic investigations of major constituents to bridge experimental and clinical findings; (IV) rigorously designed clinical trials to confirm efficacy and safety; and (V) establishment of standardized cultivation, harvesting, and quality control protocols to ensure consistency. Expanding its application as a functional food or nutraceutical may further integrate its traditional use with evidence-based healthcare.

## Figures and Tables

**Table 1:** Chemical constituents in *G. affine*.

NO.	Classify	Chemical Compound	Plant Part	Isolation/Analytical Method	Reference
1	Flavonoids	Apigenin	Dried, whole herb	Isolated (silica gel, MCI gel, RP-18, Sephadex LH-20); MS, NMR	[15]
2		Apigenin 4′-O-*β*-D-glucopyranoside	Fresh flowers	Isolated (Polyamide, Sephadex LH-20); UV, IR, NMR, acid hydrolysis, TLC	[15]
3		Apigenin 7-glucoside	Dried, aerial parts with flowers	Extracted with 70% MeOH; Identified by UPLC-Q-Exactive Orbitrap MS with authentic standard	[16]
4		Apigenin 4′-O-*β*-D-(6′′-E-caffeoyl)-glucopyranoside	Dried, whole herb	Isolated (MCI gel, Sephadex LH-20, RP-18); HRESIMS, IR, 1D NMR, 2D NMR	[15]
5		Apigenin 7-O-*β*-D-(6′′-E-caffeoyl)-glucopyranoside	Dried, whole herb	Isolated (silica gel, MCI gel, RP-18, Sephadex LH-20); MS, NMR, comparison with the literature	[15]
6		Apigenin-7-O-*β*-D-glucuronic acid methylester	Dried, aerial parts	Isolated (silica gel, Sephadex LH-20); 1D NMR, UV	[17]
7		Luteolin	Dried, whole herb	Isolated (silica gel, MCI gel, RP-18, Sephadex LH-20); MS, NMR	[15]
8		Luteolin 4′-O-*β*-D-glucopyranoside	Dried, whole herb	Isolated (silica gel, MCI gel, RP-18, Sephadex LH-20); MS, NMR, comparison with the literature	[15]
9		Luteolin 4′-O-*β*-D-(6′′-*E*-caffeoyl)-glucopyranoside	Dried, whole herb	Isolated (MCI gel, Sephadex LH-20, RP-18); HRESIMS, IR, 1D NMR, 2D NMR	[15]
10		luteolin-7-O-glucoside	Whole herb	Synthesized by recombinant PaUGT23 in vitro; identified by LC-MS	[18]
11		luteolin-7-O-*β*-D-glucopyranosyl-(1→6)-[(6′′′-O-caffeoylquinic)-*β*-D-glucopyranoside]	Whole herb	Isolated (silica gel, macroporous resin, Sephadex LH-20); ESI-MS, 1H NMR, 13C NMR, comparison with the literature	[19]
12		6-Hydroxyluteolin 7-*O*-*β*-D-glucopyranoside	Dried, whole herb	Isolated (silica gel, MCI gel, RP-18, Sephadex LH-20); MS, NMR, comparison with the literature	[15]
13		Acacetin	n/a	n/a	[17]
14		Acacetin 7-*O*-rutinoside	Dried, whole herb	Isolated (Sephadex LH-20, crystallization); MS, NMR, comparison with the literature	[15]
15		5-Hydroxy-4′,7-dimethoxyflavone	Dried, whole herb	Isolated (silica gel, Sephadex LH-20); MS, NMR, comparison with the literature	[15]
16		5-Hydroxy-6,7,3′,4′-tetramethoxyflavone	Powdered, whole plants	Extracted with 95% EtOH, isolated (silica gel column chromatography, Sephadex LH-20); ESI-MS, 1H NMR, 13C NMR, comparison with the literature	[20]
17		6-Hydroxyluteolin-7-O-*β*-D-glucopyranoside	Dried, whole herb	Isolated (RP-18, Sephadex LH-20); MS, NMR, comparison with the literature	[17]
18		5,7,4′-Trihydroxy-6,3′-methoxyflavonoid-7-O-*β*-D-(6′′-O-caffeoyloxy)-glucopyranoside	Dried, aerial parts	Isolated (polyamide, Sephadex LH-20, HPLC); HRESIMS, 1D NMR, 2D NMR	[17]
19		Eupatilin	Powdered, whole herb	Extracted with 95% EtOH, isolated (silica gel column chromatography, Sephadex LH-20); ESI-MS, 1H NMR, 13C NMR, comparison with the literature	[20]
20		Chrysin	Dried, whole herb	Extracted with 95% EtOH, isolated (silica gel column chromatography, ODS column chromatography); 1H NMR, 13C NMR, comparison with the literature	[21]
21		Wogonin			[21]
22		5-Demethyltangeretin	n/a	Synthesized from tangeretin by selective demethylation; 1H NMR, EIMS	[22]
23		Tangeretin	n/a	Isolated from Citrus aurantium peel (hexane extract); 1H NMR, EIMS	[22]
24	Flavonols	Astragalus	Dried, aerial parts with flowers	Extracted with 70% MeOH; Identified by UPLC-Q-Exactive Orbitrap MS with authentic standard	[16]
25		Kaempferol	Dried, whole herb	Isolated (silica gel, MCI gel, RP-18, Sephadex LH-20); MS, NMR	[15]
26		Kaempferol-3-O-*β*-D-glucopyranoside	n/a	n/a	[17]
27		Kaempferol 3-O-*β*-D-(6′′-O-coumarin)-glucopyranosid	n/a	n/a	[17]
28		Quercetin	Dried, whole herb	Isolated (silica gel, MCI gel, RP-18, Sephadex LH-20); MS, NMR	[15]
29		Quercetin-3-O-*β*-D-glucopyranoside	Dried, whole herb	Extracted with 95% EtOH, isolated (silica gel column chromatography, Sephadex LH-20, RP-18); 1H NMR, 13C NMR, MS, comparison with the literature	[23]
30		Quercetin 4′-*O*-*β*-D-glucopyranoside	Dried, whole herb	Isolated (Sephadex LH-20, RP-18); MS, NMR, comparison with the literature	[15]
31		Quercetin 4′-*O*-*β*-D-(6′′-*E*-caffeoyl)-glucopyranoside	Dried, whole herb	Isolated (MCI gel, Sephadex LH-20, RP-18); HRESIMS, IR, 1D NMR, 2D NMR	[15]
32		Quercetin 7-*O*-*β*-D-glucuronide	Dried, whole herb	Isolated (Sephadex LH-20, RP-18); MS, NMR	[15]
33		Quercetin 3-O-robinobioside	Whole herb	Extracted with 50% EtOH, purified by AB-8 macroporous resin, identified by LC-ESI-MS/MS, comparison with the literature	[24]
34		Hyperoside	Dried stems and leaves	Extracted with 60% EtOH, identified by UPLC-MS/MS with authentic standard	[25]
35		Quercetin 3-rutinoside 7-glucoside	Whole herb	Isolated (silica gel, macroporous resin, Sephadex LH-20); 1H NMR, 13C NMR, comparison with the literature	[19]
36		tetramethoxyquercetin	n/a	n/a	[22]
37		Quercetin 3-O-*β*-D-galactopyranoside-4′-O-*β*-D-glucopyranoside	Leaves	Isolated (Sephadex LH-20, HPLC); FABMS, 1D NMR, 2D NMR	[17]
38		Rhamnetin	n/a	n/a	[17]
39		Quercimeritrin	Dried, whole herb	Isolated (RP-18, Sephadex LH-20); MS, NMR, comparison with the literature	[15]
40		Rutin	Powdered whole plants	Extracted with 95% EtOH, isolated (silica gel column chromatography, Sephadex LH-20); ESI-MS, 1H NMR, 13C NMR, comparison with the literature	[20]
41		Quercetagetin	Dried, whole herb	Isolated (RP-18, Sephadex LH-20); MS, NMR	[15]
42		Quercetagetin 7-*O*-*β*-D-glucopyranoside			[15]
43		Isorhamnetin	Dried, whole herb	Isolated (silica gel, MCI gel, RP-18, Sephadex LH-20); MS, NMR	[15]
44		isorhamnetin-7-O-*β*-D-glucopyranoside	Dried, whole herb	Extracted with 80% EtOH, isolated (silica gel, Sephadex LH-20, RP-18); ESI-MS, 1H NMR, 13C NMR, comparison with the literature	[26]
45		scutellarein-7-O-*β*-D-glucoside			[26]
46		Gnaphaliin B	Dried, whole herb	Isolated (silica gel, Sephadex LH-20); MS, NMR, comparison with the literature	[15]
47		3,5-Dihydroxy-6,7,8,4′-tetramethoxyflavone			[15]
48		5, 3′-Dihydroxy-3, 6, 7, 8, 4′-pentamethoxyflavone	Aerial parts	Extracted with 60% EtOH, isolated (silica gel, RP-18, Sephadex LH-20, preparative HPLC); ESI-MS, 1H NMR, 13C NMR, comparison with the literature	[27]
49		5-Hydroxy-3,7,8-trimethoxyflavone	Dried, whole herb	Isolated (silica gel, Sephadex LH-20); MS, NMR, comparison with the literature	[15]
50		5-Hydroxy-3,6,7,8-tetramethoxyflavone	Fresh, whole herb	Isolated (silica gel, Sephadex LH-20); 1D NMR, EIMS, UV, IR	[15]
51		5-Hydroxy-3,6,7,8,4′-pentamethoxyflavone	Dried, whole herb	Isolated (silica gel, Sephadex LH-20); MS, NMR	[15]
52		5-Hydroxy-3,6,7,8,3′,4′-hexamethoxyflavone			[15]
53		Gnaphaliin A	Dried, whole herb	Isolated (silica gel, Sephadex LH-20); MS, NMR, comparison with the literature	[15]
54		5,7-Dihydroxy-3,6,8-trimethoxyflavone	Dried, whole herb	Isolated (silica gel, Sephadex LH-20); MS, NMR	[15]
55		5,7-Dihydroxy-3,8,4′-trimethoxyflavone	Dried, whole herb	Isolated (silica gel, Sephadex LH-20); MS, NMR, comparison with the literature	[15]
56		5,7-Dihydroxy-3,8,3′,4′-tetramethoxyflavone			[15]
57		5,6-Dihydroxy-3,7-dimethoxyflavone	Fresh, whole herb	Isolated (silica gel, Sephadex LH-20); 1D NMR, EIMS, UV, IR	[15]
58		5,7-Dihydroxy-3,8 -dimethoxyflavone	Aerial parts	Extracted with 60% EtOH, isolated (silica gel, RP-18, Sephadex LH-20, preparative HPLC); ESI-MS, 1H NMR, 13C NMR, comparison with the literature	[28]
59		5, 4′-Dihydroxy-3, 7,8-trimethoxyflavone	n/a	n/a	[27]
60		5,8-dihydroxy-3,6,7-trimethoxyflavone	Dried, aerial parts	Isolated (silica gel, Sephadex LH-20); HRMS, UV, 1H NMR, chemical derivatization	[17]
61		5,8-Dihydroxy-3,6,7,4′-tetramethoxyflavonoid	n/a	n/a	[17]
62		3, 5,7-Trihydroxy-8-methoxyflavone	n/a	n/a	[27]
63		Calycopterin	n/a	n/a	[27]
64		Galangin	n/a	n/a	[27]
65	Dihydroflavonids	Dihydroapigenin	Dried, aerial parts	Isolated (silica gel, ODS, Sephadex LH-20); 1H NMR, 13C NMR, UV, comparison with the literature	[29]
66		Naringenin-7-O-*β*-D-(6′′-E-caffeoyl)-glucopyranoside	n/a	n/a	[17]
67		Taxifolin	n/a	n/a	[17]
68	Chalcones	4,4′,6′-Trihydroxy-2′-methoxychalcone	Fresh, whole herb	Isolated (silica gel, Sephadex LH-20); 1D NMR, EIMS, UV, IR	[15]
69		Gnaphalin	n/a	n/a	[15]
70		2′,4′-Dihydroxy-4,6′-dimethoxychalcone	n/a	n/a	[22]
71		2′-Hydroxy-4,4′,6′-trimethoxychalcone	n/a	n/a	[22]
72		4,2′,4′,6′-Tetramethoxychalcone	n/a	n/a	[22]
73		2′,4,4′-trihydroxy-6′-methoxychalcone	Fresh flowers	Isolated (polyamide column chromatography); UV, 1H NMR, acid hydrolysis, methylation	[17]
74		2′,4,4′-trihydroxy-6′-methoxychalcone-4-glucopyranoside			[17]
75	Triterpenes	α-Amyrin	Dried, whole herb	Isolated (silica gel column chromatography, Sephadex LH-20); ESI-MS, 1H NMR, 13C NMR, comparison with the literature	[21]
76		α-Amyrin acetate	Dried, whole herb	Extracted with 95% EtOH, isolated (silica gel column chromatography, ODS column chromatography); 1H NMR, 13C NMR, comparison with the literature	[21]
77		Taraxasterol acetate	Dried, whole herb	Isolated (silica gel column chromatography, Sephadex LH-20); ESI-MS, 1H NMR, 13C NMR, comparison with the literature	[29]
78		Taraxasterol	n/a	n/a	[29]
79		β-Amyrin	Dried, whole herb	Extracted with 95% EtOH, isolated (silica gel column chromatography, ODS column chromatography); 1H NMR, 13C NMR, comparison with the literature	[21]
80		β-Amyrin acetate			[21]
81		Betulinic acid	Whole herb	Isolated (silica gel, Sephadex LH-20); MS, NMR	[30]
82		Betulonic acid	n/a	n/a	[17]
83		Ursolic acid	Dried, whole herb	Extracted with 95% EtOH, isolated (silica gel column chromatography, ODS column chromatography); 1H NMR, 13C NMR, comparison with the literature	[21]
84		19α-Ursolic acid			[21]
85		Oleanolic acid			[21]
86		2α, 3α, 19α-trihydroxy-28-norurs-12ene			[21]
87		lupeone	n/a	n/a	[29]
88		Friedelin	n/a	n/a	[29]
89		Squalene	Whole herb	Extracted with MeOH:H_2_O (4:1), identified by UPLC-QE-MS, comparison with self-built database	[31]
90		Faradiol 3-O-palmitate	Whole herb	Isolated (silica gel column chromatography); ESI-MS, 1H NMR, 13C NMR, comparison with the literature	[19]
91	Diterpenes	Kaurenoic acid	Whole herb	Extracted with MeOH:H_2_O (4:1), identified by UPLC-QE-MS, comparison with self-built database	[31]
92		Ent-pimara-8(14),15-dien-3a,19-diol	Dried, powdered whole herb	Isolated (silica gel, preparative TLC); MS, NMR, comparison with the literature	[17]
93		Ent-pimara-8(14),15-dien-3α-ol	Dried, powdered whole herb	Isolated (silica gel, preparative TLC); 1D NMR, 2D NMR, MS, comparison with the literature	[17]
94		Ent-pimara-8(14),15-dien-19-ol	Dried, powdered whole herb	Isolated (silica gel, preparative TLC); MS, NMR, comparison with the literature	[17]
95		Ent-Kaur-16-en-19-oic acid	Air-dried leaves	Isolated (silica gel column chromatography, crystallization); MS, NMR, comparison with the literature	[17]
96		(−)-16-Kaurene-19-oic acid			[17]
97		Zoapatlin	Aerial parts	Extracted with 90% EtOH, identified by UHPLC-Q-TOF/MS, comparison with the literature	[17]
98		Ent-3β-hydroxykaur-16-en-19-oic acid	n/a	n/a	[17]
99		(−)-11β-Acetoxy-16-kaurene-19-oic acid	Air-dried leaves	Isolated (silica gel column chromatography); MS, NMR, comparison with the literature	[17]
100	Sesquiterpenes	δ-Cadinene	Dried, whole herb	Extracted by steam distillation, identified by GC-MS, comparison with NIST library	[32]
101		β-selinene			[32]
102		Caryophyllene			[32]
103		Aromadendrene			[32]
104		1,6,10-Dodecatriene			[32]
105		α-Caryophyllene			[32]
106		Caryophyllene oxide			[32]
107		Corchoionol C	Dried, whole herb	Extracted with 80% EtOH, isolated (silica gel, Sephadex LH-20); ESI-MS, 1H NMR, 13C NMR, comparison with the literature	[33]
108		Nerolidol	Fresh, whole herb	Extracted by steam distillation, identified by GC-MS, comparison with NIST library	[34]
109		γ-Cadinene	Dried, whole herb	Extracted by steam distillation, identified by GC-MS, comparison with NIST library	[17]
110		(−)-*β*-Elemene	Whole herb	Extracted by simultaneous distillation extraction, identified by GC-MS, comparison with NIST/WILEY library	[17]
111		α-Gurjunene			[17]
112		Trans-Caryophyllene	n/a	n/a	[17]
113		α-elemol	Whole herb	Extracted by simultaneous distillation extraction, identified by GC-MS, comparison with NIST/WILEY library	[17]
114		γ-Gurjunene	Fresh, whole herb	Simultaneous distillation extraction (SDE) with diethyl ether, GC-MS analysis, identified by Wiley275 library and literature comparison	[17]
115		6,10,14-trimethyl-2-Pentadecanone	Whole herb	Extracted by steam distillation, identified by GC-MS, comparison with WILEY library	[17]
116		1,5,5,8-Tetramethyl-12-oxabicyclo[9.1.0]dodeca-3,7-diene			[17]
117	Monoterpenes	Eugenol	n/a	n/a	[17]
118		α-Terpineol	n/a	n/a	[17]
119		Linalool	n/a	n/a	[17]
120		p-cymene	n/a	n/a	[17]
121		m-cymene	Whole herb	Extracted by simultaneous distillation extraction, identified by GC-MS, comparison with WILEY library	[17]
122		Pulegone	Whole herb	Extracted by steam distillation, identified by GC-MS, comparison with NIST library	[17]
123	Phytosterols	β-Sitosterol	Dried, whole herb	Isolated (silica gel, Sephadex LH-20); TLC with authentic standard	[15]
124		(20*R*)-Cholest-4-en-3-one	Dried, whole herb	Isolated (silica gel, Sephadex LH-20); MS, NMR, comparison with the literature	[15]
125		3*β*-Hydroxy-stigmast-5,22-dien-7-one			[15]
126		Stigmasterol	Dried, whole herb	Extracted with 95% EtOH, isolated (silica gel column chromatography, ODS column chromatography); 1H NMR, 13C NMR, comparison with the literature	[21]
127		α-Spinasterol	Dried, aerial parts	Isolated (silica gel, ODS, Sephadex LH-20); 1H NMR, 13C NMR, comparison with the literature	[29]
128		Daucosterol	Dried, whole herb	Extracted with 80% EtOH, isolated (silica gel, Sephadex LH-20); ESI-MS, 1H NMR, 13C NMR, comparison with the literature	[33]
129		3β,hydroxy-stigmast-5,22-dien-7-one	Dried, whole herb	Isolated (silica gel column chromatography, Sephadex LH-20); ESI-MS, 1H NMR, 13C NMR, comparison with the literature	[30]
130		Stigmasta-4,22-dien-3-one	n/a	n/a	[17]
131	Anthraquinones	Emodin	Dried, whole herb	Isolated (silica gel, Sephadex LH-20); MS, NMR, comparison with the literature	[15]
132		Physcion			[15]
133	Phenolic acids	Ethyl protocate	Dried, whole herb	Isolated (silica gel, MCI gel, Sephadex LH-20, ODS); 1H NMR, 13C NMR, MS	[17]
134		Isovanillic acid	Dried, whole herb	Isolated (silica gel column chromatography, HPLC); 1H NMR, 13C NMR, MS	[17]
135		3′,5-dihydroxy-2- (4-hydroxybenzyl) -3-methoxybibenzyl	Whole herb	Isolated (silica gel, Sephadex LH-20, preparative TLC); ESI-MS, 1H NMR, 13C NMR, comparison with the literature	[17]
136		Methyl p-hydroxycinolinate glucoside			[17]
137		Acteoside			[17]
138		Coniferylaldehyde			[19]
139		Isovanillin	Dried, whole herb	Extracted with 95% EtOH, isolated (silica gel column chromatography, ODS column chromatography); 1H NMR, 13C NMR, comparison with the literature	[21]
140		Everlastoside L	Dried, whole herb	Extracted with 80% EtOH, isolated (silica gel, Sephadex LH-20, RP-18); ESI-MS, 1H NMR, 13C NMR, comparison with the literature	[26]
141		Protocatechuic acid	Dried, aerial parts	Extracted with 80% MeOH, identified by HPLC with authentic standard	[35]
142		Ethyl 3,4-dihydroxybenzoate	Aerial parts	Extracted with 60% EtOH, isolated (silica gel, RP-18, Sephadex LH-20, preparative HPLC); ESI-MS, 1H NMR, 13C NMR, comparison with the literature	[28]
143		p-Coumaric acid			[28]
144		ferulic acid	Whole herb	Extracted with 80% MeOH, identified by LC-MS/MS, comparison with the literature	[36]
145		caffeic acid	Dried, aerial parts	Extracted with 80% MeOH, identified by HPLC with authentic standard	[35]
146		Methyl caffeate	Aerial parts	Extracted with 60% EtOH, isolated (silica gel, RP-18, Sephadex LH-20, preparative HPLC); ESI-MS, 1H NMR, 13C NMR, comparison with the literature	[27]
147		Phenethyl caffeate			[28]
148		1-O-caffeoyl-*β*-D-glucopyranose			[27]
149		Gallic acid	Dried, Aerial parts with flowers	Extracted with 70% MeOH; Identified by UPLC-Q-Exactive Orbitrap MS with authentic standard	[16]
150		quinic acid			[16]
151		Chlorogenic acid	Dried, aerial parts	Extracted with 80% MeOH, identified by HPLC with authentic standard	[35]
152		3-O-caffeoylquinic acid	Whole herb	Extracted with 80% MeOH, identified by LC-MS/MS, comparison with the literature	[36]
153		4-O-caffeoylquinic acid			[36]
154		5-O-caffeoylquinic acid			[36]
155		1,3-di-*O*-Caffeoylquinic acid	Aerial parts	Extracted with 60% EtOH, isolated (silica gel, RP-18, Sephadex LH-20, preparative HPLC); ESI-MS, 1H NMR, 13C NMR, comparison with the literature	[28]
168	Alkaloids	Scopolamine	n/a	n/a	[17]
169		2-Hydroxy-N-(4-hydroxyphenyl)-benzamide	Dried, aerial parts with flowers	Extracted with 70% MeOH; Identified by UPLC-Q-Exactive Orbitrap MS with authentic standard	[16]
170		longumoside A	Whole herb	Isolated (silica gel, Sephadex LH-20, preparative TLC); HR-ESI-MS, 1H NMR, 13C NMR, comparison with the literature	[19]
171		grossamide K	Whole herb	Isolated (silica gel, Sephadex LH-20, preparative TLC); ESI-MS, 1H NMR, 13C NMR, comparison with the literature	[19]
172		anabellamide			[19]
173		Patriscabratine	Dried, whole herb	Extracted with 95% EtOH, isolated (silica gel column chromatography, Sephadex LH-20); ESI-MS, 1H NMR, 13C NMR, comparison with the literature	[21]
174		aurantiamide acetate	Dried, whole herb	Extracted with 95% EtOH, isolated (silica gel column chromatography, ODS column chromatography); 1H NMR, 13C NMR, comparison with the literature	[21]
175	Others	Phloroglucinol	Dried, whole herb	Extracted with 80% EtOH, isolated (silica gel, Sephadex LH-20); IR, MS, NMR, comparison with the literature	[17]
176		3-methoxyphenol1-O-α-L-rhamnopyranosyl-4- (1→6)-O-*β*-D-glucopyranoside	Whole herb	Isolated (silica gel, Sephadex LH-20, preparative TLC); ESI-MS, 1H NMR, 13C NMR, comparison with the literature	[17]
177		7-O-(β-D-glucopyranosyl)-5-hydroxyisobenzofuran-1(3H)-one	Aerial parts	Extracted with 95% EtOH, isolated (silica gel column chromatography); ESI-MS, 1H NMR, 13C NMR, comparison with the literature	[17]
178		4-(3′,4′-dimethoxyphenyl)-3-methyl-butyl-3-ene-2-one			[17]
179		5,7-dihydroxyl-isobenzofuran-1(3H)-one			[17]
180		1-Hexacosanol	Dried, whole herb	Isolated (silica gel column chromatography, HPLC); 1H NMR, 13C NMR, MS, comparison with the literature	[17]
181		Benzeneacetaldehyde	Whole herb	Extracted by simultaneous distillation extraction, identified by GC-MS, comparison with WILEY library	[17]
182		1-Tetratriacontanol	Dried, whole herb	Extracted with 80% EtOH, isolated (silica gel column chromatography, Sephadex LH-20); ESI-MS, 1H NMR, comparison with the literature	[17]
183		Diethyl phthalate	Whole herb	Extracted by simultaneous distillation extraction, identified by GC-MS, comparison with WILEY library	[17]
184		Myristicin aldehyde	Dried, whole herb	Extracted by steam distillation, identified by GC-MS, comparison with NIST library	[17]
185		N-butyl-isobutyl terephthalate	Aerial parts	Extracted with 95% EtOH, isolated (silica gel column chromatography); 1H NMR, 13C NMR, comparison with the literature	[17]
186		2,3,5,4′-tetrahydtoxysilbene-2-O-*β*-D-glucopyranoside	Dried, whole herb	Extracted with 80% EtOH, isolated (silica gel, Sephadex LH-20, ODS); ESI-MS, 1H NMR, 13C NMR, comparison with the literature	[17]
187		valene-1(10)-ene-8, 11-diol	Whole herb	Isolated (silica gel, Sephadex LH-20, preparative TLC); ESI-MS, 1H NMR, 13C NMR, comparison with the literature	[19]
188		Isoverbascoside			[19]
189		3-methoxyphenol-1-O-α-L-rhamnopyranosyl-(1→6)-O-*β*-D-glucopyranoside			[19]
190		4-O-D-glucopyranosyl-p-coumaric acid methyl ester			[19]
191		Gnaphaffine A	Dried, whole herb	Extracted with 80% EtOH, isolated (silica gel, Sephadex LH-20, RP-18); ESI-MS, 1H NMR, 13C NMR, comparison with the literature	[26]
192		Gnaphaffine B			[26]
193		Dibutyl phthalate	Whole herb	Extracted by steam distillation, identified by GC-MS, comparison with WILEY library	[27]
194		2-Ethylhexyl phthalate	n/a	n/a	[27]
195		Ethyl 3, 4-dihydroxybenzoate	Aerial parts	Extracted with 60% EtOH, isolated (silica gel, RP-18, Sephadex LH-20, preparative HPLC); ESI-MS, 1H NMR, 13C NMR, comparison with the literature	[27]
196		Methylparaben			[27]
197		Adenosine	n/a	n/a	[27]
198		5′-Deoxy-adenosine	n/a	n/a	[27]
199		Adenine	Dried, whole herb	Extracted with 80% EtOH, isolated (silica gel, Sephadex LH-20, RP-18); ESI-MS, 1H NMR, 13C NMR, comparison with the literature	[26]
200		Octadecanoic acid	Whole herb	Extracted by steam distillation, identified by GC-MS, comparison with WILEY library	[27]
201		n-hexacosanic acid	Dried, whole herb	Isolated (silica gel column chromatography, Sephadex LH-20); ESI-MS, 1H NMR, 13C NMR, comparison with the literature	[30]
202		Tetradecanal	Dried, whole herb	Extracted by steam distillation, identified by GC-MS, comparison with NIST library	[32]
203		Dodecanal			[32]
204		Pentadecanone			[32]
205		9,17-Octadecadienal			[32]
206		n-hexadecanoic acid			[32]
207		6,10,14(Trimethyl-5,9,13-Pentadecatrien-2-one			[32]
208		n-tetratriacontanol	Dried, whole herb	Extracted with 80% EtOH, isolated (silica gel, Sephadex LH-20); ESI-MS, 1H NMR, 13C NMR, comparison with the literature	[33]
209		glyceryl monopalmitate			[33]
210		9,16-dioxo-10,12,14-octadeca-trienoic acid			[33]
211		Tithoniamide B			[33]
212		2,3,5,4′-Tetrahydroxy stilbene-2-O-*β*-D-glucoside			[33]
213		4′-Hydroxyacetophenone	Aerial parts	Extracted with 60% EtOH, isolated (silica gel, RP-18, Sephadex LH-20, preparative HPLC); ESI-MS, 1H NMR, 13C NMR, comparison with the literature	[28]
214		4′-hydroxydehydrokawain			[28]
215		3-(4′-formylphenoxy)-4-methoxybenzaldehyde			[28]
216		desmethylyangonine-4′-glucopyranoside			[28]

Table notes: n/a (not applicable or not available).

**Table 2:** Pharmacological effects and mechanism of action of *G. affine*.

Biological Activity	Compound	Subject	Parameter	Effects/Mechanisms	References
Antibacterial	Flavonoids	In vitro, bacterial suspension	24 h	Antibacterial (*S. aureus, Salmonella, B. subtilis, E. coli*)	[37]
	Flavonoids	In vitro, bacterial suspension	24 h	Antibacterial (*E. coli, B. subtilis, S. aureus*), concentration dependence	[38]
	Flavonoids, Flavonoids -Zn	In vitro, viruses	24 h	Damage to bacterial cell membranes and oxidative breakdown of bacterial DNA, stronger inhibition against Gram-positive bacteria	[39,40]
Cough expectorant	Water extracts	In vivo, mice, guinea pigs	0.2 mL/10 g per day for 7 Days	Cough suppressant and expectorant; tracheal phenol red excretion ↑	[41]
	Alcohol extracts	In vivo, COPD rat	0.1 g/100 g per day for 7 Days	Lung inflammatory infiltration ↓; BALF IL-8 ↓, TNF-α ↓; lung IL-8 and TNF-α mRNA ↓	[42]
	Alcohol extracts	In vivo, COPD rat	0.75, 1.5, 3 g/kg per day for 14 Days	Serum CAT ↑, GSH ↑; lung SOD ↑, MDA ↓; Nrf2, HO-1, NQO1 mRNA ↑ and protein ↑; modulated gut microbiota	[43]
	Flavonoids	In vivo, CB mice	0.2 mL/10 g per day for 7 Days	Relieve cough, resolve phlegm and asthma	[44]
Anti-inflammatory	Alcohol extracts	In vivo, AA rats	0.4, 0.80, 1.60 g/kg per day for 21 Days	PGE2, IL-1 and TNF-α ↓, CD_4_^+^/CD_8_^+^ ↑	[45]
	Alcohol extracts	In vivo, GA rats	0.40, 0.80, 1.60 g/kg per day for 8 Days	UA, IL-1β, TNF-α, NALP3, Caspase-1 and ASC ↓, and XOD ↓	[46]
	Alcohol extracts	In vivo, zebrafish viral pneumonia model	125, 250, 500 µg/ml	Inflammatory infiltration ↓, IL-1β and TNF-α ↓	[47]
	Extracts	In vivo, OA rats	400, 800, 1600 mg/kg	PERK/eIF2α/CHOP signal pathway ↓	[48]
	Alcohol extracts	In vivo, CIA rats; in vitro, NR8383 cell	75, 150, 300 mg/kg; 50, 100, 200 µg/mL	Regulate the NF-κB signal pathway, NO, TNF-α, IL-1β, COX-2 ↓	[49]
	Flavonoids	In vivo, mice pain model	25, 50, 100 mg/kg per day for 7 Days	TNF-α, IL-6, NO ↓ and TNF-α, IL-6, iNOS ↓	[50]
	Flavonoids	In vivo, COPD rats	50 mg/kg per day for 7 Days	Caspase-3, IL-1β, IL-6 and TNF-α ↓	[51]
Antioxidant	Quercetin	In vitro, H_2_O_2_ induced Caco-2 cell	10, 20, 30, 40 and 50 µg/mL	ABTS, DPPH, superoxide, hydroxyl radicals scavenging ↑, lipid peroxidation inhibition ↑, Scavenge ·OH and O_2_^−^	[52]
	Polysaccharide	In vitro, hydroxyl and superoxide anion radical scavenging assay	-	Scavenge ·OH and O_2_^−^, concentration range 0.4–2.0 mg showed dose-dependent activity	[53]
	Polyphenol, flavonoids	In vitro, H_2_O_2_ induced H9c2 cell	50 mg/L	ROS, MDA ↓, SOD ↑, Nrf2 ↑, cleaved-caspase 3 ↓; p-AKT/AKT ↑, PI3K/AKT/GSK-3β ↑	[54]
	Flavonoids	In vitro, H_2_O_2_-induced HepG2 cells	2, 5, 10 μg/mL	ROS ↓, LDH ↓, SOD and CAT ↑, GSH ↑, Nrf2, HO-1, and NQO-1 ↑, and keap1 ↓	[55]
	Flavonoids	In vivo, DM mice	50, 100 mg/kg per day for 21 Days	Serum and liver: SOD ↑, CAT ↑, GSH-Px ↑(partial), T-AOC ↑, MDA ↓	[56]
	Flavonoids	In vitro, total antioxidant, superoxide anion radical, DPPH radical, hydroxyl radical scavenging assay and Fe ^2+^ induced yolk lipoprotein peroxidation assay	0.10, 0.15, 0.20, 0.25, 0.30 mg/mL	Total antioxidant capacity ↑ (dose-dependent); hydroxyl radical scavenging ↑ (stronger than VC); superoxide anion inhibition ↑, DPPH scavenging ↑, Fe^2+^-induced lipid peroxidation inhibition ↑	[57]
	Flavonoids	In vitro, scavenges DPPH radicals, hydroxyl radicals, nitrite assay	-	DPPH scavenging ↑, hydroxyl radical scavenging ↑, nitrosamine synthesis blocking ↑, NaNO_2_ scavenging ↑	[58]
	Alcohol extracts	In vitro, ABTS and hydroxyl radical scavenging assay	-	Antioxidant; phenolic and flavonoid ↑ with moderate temperature, time, and power	[59]
Hypoglycemic	Alcohol extracts	In vitro, α-amylase	-	α-amylase ↓	[60]
	Flavonoids	In vivo, DM mice	50 mg/kg per day for 7 Days	glucose tolerance ↑, TC, TG, LDL-C ↓, iglycogen and HDL-C ↑	[61]
Lowering of uric acid	Flavonoids	In vivo, HUA rats	100, 300 mg/kg per day for 21 Days	XOD ↓, SUA, Cr, BUN, SOD, CAT and MDA↓	[62]
	Extracts	In vitro, XOD	-	XOD ↓	[63]
	Luteolin-4′-O-glucoside	In vivo, HUA mice	100, 200, 400 mg/kg per day for 7 Days	Affect renal mGLUT9 and mURAT1, XOD ↓, IL-1β and TNF-α ↓	[63]
	Extracts	In vivo, HN rats	450 mg/kg for 21 Days	CYP2C11 ↓	[64]
Hepatoprotection	Total flavonoids	In vivo, alcohol-induced liver injury mouse	75, 150, 300 mg/kg per day for 30 Days	oxidative stress ↓	[65]
	Alcohol extracts	In vivo, HIRI rats	0.40, 0.80, 1.60 g/kg per day for 28 Days	ALT, AST, LDH, NF-κB, p65 ↓, SOD, GSH-Px and GST ↑	[46]
	Total flavonoids	In vivo, APAP-induced ALI mice	100, 200, 400 mg/kg per day for 14 Days	ALT, AST, TNF-α, IL-6, MDA, ROS, 4-HNE ↓, liver injury ↓, Nrf2, HO-1, SLC7A11 and GPX-4 ↑, keap1 ↓and hepatocyte ferroptosis ↓	[66]
Antitumor	Water extracts	In vivo, bladder cancer rats	2, 4, 8 mg/ml	bladder cancer cell death ↑ and survivin in bladder cancer tissues ↓	[67]
	Water extracts	In vivo, lung cancer rats	2, 4, 8 mg/ml	the apoptosis of lung cancer cells ↑ and survivin in lung cancer tissues ↓	[68]
Eye protection	Dicaffeoylquini-c acids	In vitro, CECs; in vivo, DED mice	5–500 µg/mL for 7 Days	the corneal epithelial apoptosis ↓, tear production ↑ and inflammation ↓	[69]

Table notes: AA (adjuvant arthritis), ABTS (2,2′-Azinobis-(3-ethylbenzthiazoline-6-sulphonate)), AKT (protein kinase B), ALI (acute liver injury), ALT (alanine aminotransferase), APAP (Acetaminophen), ASC (apoptosis-associated speck-like protein), AST (aminotransferase), BALF (bronchoalveolar lavage fluid), BUN (blood urea nitrogen), CAT (catalase), CB (chronic bronchitis), CD (cluster of differentiation), CECs (corneal epithelial cells), CHOP (C/EBP homologous protein), CIA (collagen-induced arthritis), COPD (chronic obstructive pulmonary disease), COX-2 (cyclooxygenase-2), Cr (creatinine), CYP2C11 (cytochrome P450 2C11), DED (dry eye disease), DM (diabetes mellitus), DNA (deoxyribo nucleic acid), DPPH (2,2-diphenyl-1-picrylhydrazyl), eIF2α (eukaryotic initiation factor 2α), GA (gouty arthritis), GPX-4 (glutathione peroxidase 4), GSH (glutathione), GSH-Px (glutathione peroxidase), GSK-3β (glycogen synthase kinase-3β), GST (glutathione s-transferase), HDL-C (high-density lipoprotein cholesterol), HIRI (hepatic ischemia–reperfusion injury), HN (hyperuricemic nephropathy), 4-HNE (4-hydroxynonenal), HO-1 (heme oxygenase-1), H_2_O_2_ (hydrogen peroxide), HUA (hyperuricemia), iNOS (inducible nitric oxide synthase), IL-1 (interleukin-1), IL-1β (interleukin-1β), keap1 (Kelch-like ECH-associated protein 1), LDH (lactate dehydrogenase), LDL-C (low-density lipoprotein cholesterol), MDA (malondialdehyde), mGLUT9 (mouse glucose transporter 9), mRNA (messenger ribonucleic acid), mURAT1 (mouse urate transporter 1), NALP3 (NACHT-LRR-PYD-containing protein-3), NF-κB (nuclear factor κB), NO (nitric oxide), NQO-1 (quinone oxidoreductase 1), Nrf2 (nuclear factor erythroid 2-related factor 2), NR 8383 (rat alveolar macrophages cell), ·OH (hydroxyl radical), p65 (nuclear factor of kappa light polypeptide gene enhancer In B-Cells 3), PERK (protein kinase r-like endoplasmic reticulum kinase), PGE2 (prostaglandin E2), PI3K (phosphatidylinositol 3-Kinase), ROS (reactive oxygen species), SOD (super oxide dismutase), SUA (blood uric acid), TC (total cholesterol), TG (triglyceride), TNF-α (tumor necrosis factor-α), UA (uric acid), XOD (xanthine oxidase), ↑: up-regulated, increase, activate, and ↓: down-regulated, decrease, inhibit.

## Data Availability

No new data were created or analyzed in this study. Data sharing is not applicable.

## Abbreviations

The following abbreviations are used in this manuscript:

AKT	protein kinase B
ALT	alanine aminotransferase
APAP	acetaminophen
BBR	benzbromarone
Bcl-2	B-cell lymphoma 2
CAT	catalase
CD	cluster of differentiation
CECs	corneal epithelial cells
CHOP	C/EBP homologous protein
COPD	chronic obstructive pulmonary disease
DNA	deoxyribo nucleic acid
DPPH	2,2-diphenyl-1-picrylhydrazyl
eIF2α	eukaryotic initiation factor 2α
GA	gouty arthritis
GSH	glutathione
GSH-Px	glutathione peroxidase
GSK-3β	glycogen synthase kinase-3β
GST	glutathione s-transferase
HIRI	hepatic ischemia–reperfusion injury
HO-1	heme oxygenase-1
HUA	hyperuricemia
IL-1β	interleukin-1β
IL-6	interleukin-6
keap1	Kelch-like ECH-associated protein 1
LDH	lactate dehydrogenase
MBC	minimum bactericidal concentration
MDA	malondialdehyde
MIC	minimum inhibitory concentration
NALP3	NACHT-LRR-PYD-containing protein-3
NF-κB	nuclear factor κB
Nrf2	nuclear factor erythroid 2-related factor 2
p65	nuclear factor of kappa light polypeptide gene enhancer in B-Cells 3
PERK	protein kinase r-like endoplasmic reticulum kinase
PGE2	prostaglandin E2
PI3K	phosphatidylinositol 3-Kinase
ROS	reactive oxygen species
SOD	super oxide dismutase
TNF-α	tumor necrosis factor-α
UA	uric acid
XOD	xanthine oxidase
